# Traditional use of medicinal plants by the Jaintia tribes in North Cachar Hills district of Assam, northeast India

**DOI:** 10.1186/1746-4269-2-33

**Published:** 2006-08-09

**Authors:** Albert L Sajem, Kuldip Gosai

**Affiliations:** 1 Department of Botany, Haflong Government College, Haflong-788819 Assam, India; 2 Department of Agricultural Engineering, North Eastern Regional Institute of Science and Technology, Nirjuli-791109, Arunachal Pradesh, India

## Abstract

The study of ethnobotany relating to any tribe is in itself a very intricate or convoluted process. This paper documents the traditional knowledge of medicinal plants that are in use by the indigenous Jaintia tribes residing in few isolated pockets of northeast India. The present study was done through structured questionnaires in consultations with the tribal practitioners and has resulted in the documentation of 39 medicinal plant species belonging to 27 families and 35 genera. For curing diverse form of ailments, the use of aboveground plant parts was higher (76.59%) than the underground plant parts (23.41%). Of the aboveground plant parts, leaf was used in the majority of cases (23 species), followed by fruit (4). Different underground plant forms such as root, tuber, rhizome, bulb and pseudo-bulb were also found to be in use by the Jaintia tribe as a medicine. Altogether, 30 types of ailments have been reported to be cured by using these 39 medicinal plant species. The study thus underlines the potentials of the ethnobotanical research and the need for the documentation of traditional ecological knowledge pertaining to the medicinal plant utilization for the greater benefit of mankind.

## Background

Plants are the basis of life on earth and are central to people's livelihoods. Tribal people are the ecosystem people who live in harmony with the nature and maintain a close link between man and environment. Indian subcontinent is being inhabited by over 53.8 million tribal people in 5000 forest dominated villages of tribal community and comprising 15% of the total geographical area of Indian landmasses, representing one of the greatest emporia of ethno-botanical wealth [1]. The Northeastern states of India that comprises of eight sister states viz. Arunachal Pradesh, Assam, Manipur, Meghalaya, Mizoram, Nagaland, Sikkim and Tripura harbors more than 130 major tribal communities of the total 427 tribal communities found in India (2001 census). In general, the tribes of North East India have been categorized into two broad ethnic communities-Khasi and the Jaintia tribes of Meghalaya, who belong to 'Monkhemar' culture of Austric dialect and the rest of the tribal groups are basically Mongoloid, who belongs to Tibeto-Burman subfamily of Tibeto-Chinese group [2-4].

The Gateway of North East India, Assam with its stunning scenic grandeur entices the adventurer and tourist alike with its verdant valleys, rolling tracts of paddy and tea, misty mountain peaks, swift rivers comprises 12.8% of the total tribal population of India (2001 census). The Census enumerates 14 hill tribes; 12 of these are tribes whose settlements go back to the days of undivided Assam and include pockets of Khasi, Jaintia, and related tribes, Garo, Naga and a clutch of Kuki and related tribes – the most numerous and significant component outside the eight plains tribes and two hill tribes. Karbis (63.8%) and Dimasa (14.4%) form the major hill tribal population of Assam (1991 census). A rich diversity of both of population and flora in the state has provided an initial advantage to its inhabitants since times immemorial for observing, and scrutinizing the rich flora and fauna for developing their own traditional knowledge. The history reveals that most of the tribal economies have been engaged in subsistence agriculture or hunting and gathering. With the passage of time, they have developed a great deal of knowledge on the use of plants and plant products in curing various ailments. They have a deep belief in their native folklore medicine for remedies and they rely exclusively on their own herbal cure.

Although different workers have documented the uses of various medicinal plants from different parts of India [3,5,10-40] information on the traditional and cultural practices of the varied tribes residing in the North Cachar Hills district of Assam is unavailable. Therefore, a need was felt to gather in-depth information on the plant species used by the Jaintia tribal group and document their traditional knowledge and cultural practices which may be under threat due to the influence of modernization.

### Study area: Jaintia group of villages

**Figure 1:**
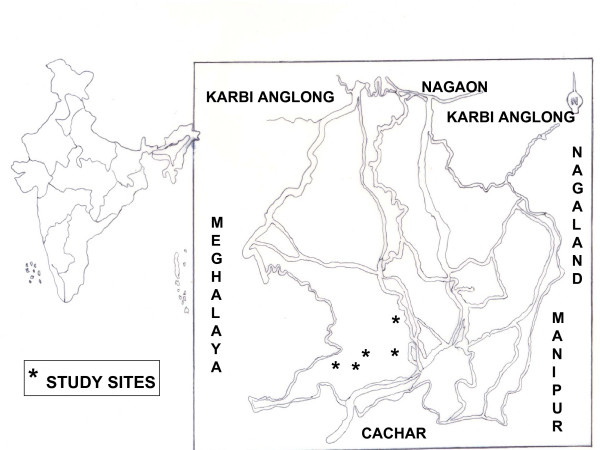
Jaintia inhabited villages of North Cachar Hills.

The Jaintias or the Pnars who bear the history of migration to about 1905 in Jatinga are among the prominent inhabitants of the North Cachar Hills (25°3'N–25°47'N latitude and 92°37'E – 93°17'E longitude) situated at the southern part of Assam and bounded by Nagaland and Manipur in the east, Cachar district of Assam in the south, Meghalaya state and the part of Karbi Anglong & Nagaon district in the north (Figure 1). They are concentrated mainly at Jatinga and the rest of the communities are scattered all over the district inhabited villages like Borolukha, Khoyong, Bandarkhal, Dimbrucherra, Harangajao, Ditokcherra, Mailongdisa etc., situated at an altitude of about 700–1100 meters. Jaintia dialect has 12 spoken forms: Jowai, Shangpung, Batau, Raliang, Sutnga, Sumer, Martiang, Barato, Rymbai, Lakadong, Mynso and Nongtalang. Jowai is the standard spoken form among all these [6].

The pattern of Jaintia villages is that of scattered settlements (village houses are distributed throughout) and homes are made of bamboo and timber. Rong Khla is the most important festival of the Jaintia tribes. Most of the people in this village are still in their traditional religion. Among their festivals include Rong Beh Dein Khlam, a festival to drive away evils. Another festival the Rong Pyrtuh is also an important one and the Phur, which is connected with the bone collection ceremony of the dead. Rong means festival and Khla means tiger, in the local dialect, so Rong Khla means the Tiger Festival.

### Methodology

A survey was carried out during 2000–2002 to collect information on the medicinal uses of plants found in the Jaintia inhabited villages of Borolukha, Khoyong, Bandarkhal, Dimbrucherra, Harangajao, Ditokcherra and Mailongdisa located in the hills (Figure 1). The above villages lie between (25°3'N – 25° 47'N latitude and 92°37'E – 93 ° 17'E longitudes) and belong to the North Cachar Hills district of Assam, northeast India. Routine methods of plant collection and herbarium techniques [7] have been followed in the study. The plants were collected in its flowering stage as far as possible, from its natural habitat.

While collecting information on ethno medicinal plants, information have been gathered from the village chiefs (Gaon Burahs), medicine man, and even local man and women and cultivators using semi-structured questionnaires. Analysis of data was made with the help of group discussions among different age classes of Jaintia villagers that include both the genders of the society. A total of 1258 villagers (759 men and 499 women) participated in the study, but only 781 (or 62% of the 1258) provided information for all the methods of data collection. The permanent sample was almost evenly split between women (n = 435, or 55.7%) and men (n = 346, or 44.3%), the average age of which was 43.9 years. Based on these information, a consensus index was determined (by calculating the percentage of informants who have quoted a given specific use of a given plant taxon) that can later be used for further scientific investigations. Some medicinal plants have also been procured which are domesticated by the local tribes for day to day use and for the treatment of common ailments.

Information about the plants were recorded with regards to their vernacular names, plant part used, process of preparation of medicine either individually or in combination with other plant parts, and mode of application and doses for the treatment of a particular disease or diseases. All the voucher specimens were identified using relevant floras and standard literature [8] and were deposited in the Department of Botany [DoB], Haflong Government College [HGC], Haflong. The collected information was analyzed, and correlation was made between different genera and species of the medicinal plants in order to understand the pattern in medicinal plant uses and occurrences.

### Results and discussion

The Jaintias of North Cachar Hills district depends on Jhum or slash and burn cultivation. Jhum is a major component of the larger agro-ecosystem that comprises of agriculture, forestry, hunting & fishing and is a land use system described as to be based on a traditional, year round, community wide, largely self contained and ritually sanctioned way of life [9]. Jaintias make sustainable use of available natural resources that includes bamboo, cane, pine and trees like A. *heterophyllus *and M. *indica *A. *chama*, A.*lakoocha*, *Garcinia *sp., *Licuala peltata*, *Sapindus mukorossi, Tectona grandis*,. *Toona ciliata, Caryota urens, Cassia siamea *etc. for different domestic as well as construction purposes. Bamboo species like *Phyllostachys bambusoides *and *Dendrocalamus hamiltonii *are also cultivated for construction of houses and other domestic uses.

The present course of investigations has revealed the usage of 39 medicinal plant species used by the Jaintias tribes from the North Cachar Hills district of Assam. The information on scientific name, local name of the plant part used to cure and method of dosage has been provided. The specimen number of the plant that has been deposited in the herbarium [DoB] of HGC has also been provided (Table 1).

**Table 1:** Medicinal plants used by Jaintia tribes of the North Cachar Hills district of Assam, northeast India.

***Scientific name (voucher specimen number and botanical family)***	***Local name***	***Part(s) used***	***Ethnomedical preparation and use (consensus index)***
*Achyranthes aspera *L. (21, Amaranthaceae)	Soh-berthid	Leaf	Pills (1–2 g each) are made out of crushed leaves and each pill is applied twice daily on boils till it heals (49%)
*Adhatoda vesica *Mill. (25, Acanthaceae)	Toh-phaileng	Flowers & Leaves	Fresh flowers and leaves are boiled in water and decoction is prepared which is consumed once in a day to cure nose bleeding, dysentery and blood vomiting (63%)
*Ageratum conyzoides *L. (32, Asteraceae)	Tuah-dain	Leaves	Crushed leaves are used directly on cuts and wounds (69%)
*Alstonia scholaris *(L.) R.Br. (41, Apocynaceae)	Gumbuthen	Bark	Fresh barks are cut into small pieces and decoction is prepared which is later filtered through a cloth, concentrated and dried in shade; out of this small pills (each of ca 1–1.5 g) are made, three pills a day (for adults) is the recommended dosage for curing asthma (75%)
*Amomum dealbatum *Roxb. (48, Zingiberaceae)	Salaphiah	Roots/Rhizome	Rhizome or roots are crushed and then fried lightly with mustard oil and is applied to cure joint pains (71%)
*Arum dioscoridis *Sibth. & Sm. (53, Araceae)	Wang-yong	Stem	Stems are crushed and the extract is applied directly to cure boils (58%)
*Asparagus racemosus *Willd. (61, Liliaceae)	Lamardoh	Leaves	Dried leaves are powdered and are taken orally to cure stomach ache and urinary disorders (83%)
*Barleria cristata *Alba (71, Acanthaceae)	Sajhia	Aerial parts	Entire plant is crushed, boiled in water and filtered; 2–3 drops of decoction is used against skin infections (56%)
*Begonia roxburghii *(Miq.) DC. (75, Bigoniaceae)	Jajau-mo	Rhizome/Bulb	It is crushed and applied on the body parts where the thorns are stuck to prevent further infection and allow it to come out by itself (80%)
*Bryophyllum calycinum *Salisb. (82, Crassulaceae)	Dawaiein	Leaves	Leaves are crushed and are applied on burns and bruises; eye sores, eye pain or eye itching twice daily (71%)
*Cassia tora *L. (103, Caeselpinaceae)	Dain-trut	Leaves, barks & roots	Leaves, barks and roots are applied externally on skin diseases such as ring worms, leprosy (52%)
*Cataranthus roseus *(L.) G. Don. (122, Apocynaceae)	Santujri-so	Leaves	Leaves are taken directly (about a handful) for diabetes and high blood pressure; 2–3 drops of this extract is poured in the nostril to cure nasal bleeding (67%)
*Centella asiatica *(L.)Urban (132, Apiaceae)	Wangrake	Whole	Decoction of leaves is used against conjunctivitis and other eye injury; crushed leaves are mixed in a cup of water with a tablespoon of salt and taken once daily for stomachic, indigestion and flatulence (78%)
*Clerodendrum grandulosum *L. (163, Verbenaceae)	Jhr-khtung	Leaves	Leaves are taken raw or are prepared along with vegetable for curing diabetes and high blood pressure (63%)
*Clerodendrum serratum *(L.) Moonb. (163, Verbenaceae)	Khr-khtung	Leaves	Whole body parts are ground with water to prepare a paste which is applied to cure fever (56%)
*Clerodendrum viscosum *Vent. (163, Verbenaceae)	Jhr-khtung	Leaves	Leaves are taken raw or are mixed with vegetable for curing diabetes, high blood pressure and asthma (82%)
*Coriandum *sativum L. (173, Umbelliferae)	Loruphi	Fruits	Dried fruits are powdered and taken orally to cure stomach ache (60%)
*Curcuma longa *L. (185, Zingiberaceae)	Chyrmit	Rhizome	Pills (1–2 g each) are made out of crushed rhizomes and each pill is taken orally before food to counter-act dyspepsia (80%)
*Cuscuta reflexa *Roxb. (199, Convolvulaceae)	Jarma	Whole	Whole plant parts are crushed and applied on the scalp to prevent premature hair fall, graying of hair and control of dandruff (79%)
*Desmodium triquetrum *(L.) DC. (221, Leguminaceae)	Yeyjur	Leaf and seeds	Leaves as well as seeds are crushed; pills (ca 1–2 g each) prepared and is used as Vermifuge- two pills daily with empty stomach is the recommended dosage (69%)
*Gossypium arboreum *L. (249, Malvaceae)	Kamphat	Seeds	Young and premature seeds are crushed; pills (ca 5–6 g each) are been prepared-one pill a day, preferably with milk is taken in empty stomach to improve memory power (83%)
*Melastoma malabathricum *L. (383, Melastomaceae)	Sarudong	Leaves/Young twigs	A handful of young premature leaves are taken raw twice daily in an empty stomach to cure dysentery (57%)
*Mikania micarantha *Kunth. (457, Asteraceae)	Jarma repuji	Leaves	Leaves are crushed; a table-spoon of the extract is taken thrice daily to cure diarrhea and dyspepsia (83%)
*Mimosa pudica *L. (463, Mimosaceae)	Klim-tchakaw	Roots	Fresh roots (ca 500 g) are crushed and soaked in (ca 500 ml) water; 100 ml of the extract is taken twice daily for curing piles (93%)
*Momordica charantia *L. (498, Cucurbitaceae)	Daipiat	Leaf and fruit	Leaves are crushed then taken orally or applied to the injured tissues for curing rabies and are also taken along with other vegetables to get rid from chest pain and other rheumatic pain (61%)
*Nicotiana tabaeccum *Viv. (535, Solanaceae)	Duma-sla	Aerial parts	Entire plant is ground and applied to the infected area thrice daily against skin infections (60%)
*Ocimum sanctum *L. (712, Lamiaceae)	Lapane	Leaves	Leaves (ca 200 g) are crushed and is later filtered through a cloth-10 ml of the extract is taken twice daily for curing stomach ache and head ache (79%)
*Oxalis corymbosa *L. (765, Oxalidaceae)	Sakhia-palleh	Whole	Entire plant is crushed and the extract is taken thrice daily to counteract dyspepsia and jaundice (85%)
*Phyllanthus niruri *L. (803, Euphorbiaceae)	Santu-plain-jarmi	Leaves & roots	Leaves (ca 500 g) are crushed and are later filtered-20 ml of the extract is taken thrice daily to cure diarrhea; roots (ca 200 g) are crushed and filtered-20 ml of the extract is taken thrice daily to cure fever (88%)
*Piper longum *L. (815, Piperaceae)	Samaran	Fruit & Roots	Crushed fruit mixed with jaggery and ginger powder is boiled (with ca 200 ml water) and is taken thrice daily before food for curing malaria; dry roots (ca 500 g) are crushed and taken with tea twice daily to cure body ache (69%)
*Plantago major *L. (880, Plantaginaceae)	Chhakur-blang	Leaves	An equal proportion of crushed leaves and raw milk (w/v) is mixed and taken in an empty stomach for almost a week to cure jaundice; leaf extract is used for curing ear ache, tooth ache and gum bleeding (73%)
*Polygonum chinense *L. (912, Polygonaceae)	Salandem	Leaves	Leaves are ground and the extract is taken thrice daily to counteract dyspepsia (79%)
*Polygonum affine *L. (912, Polygonaceae)	Jarian	Leaves	Leaves are crushed and applied on the wounds to stop bleeding (72%)
*Scoporia dulcis *L. (1028, Scrophulariaceae)	Gymbat-pdyp	Whole	Aerial parts are boiled and decoction is used for gargles; root extract (ca 200 g) is prepared and applied twice daily to prevent cavity formation (65%)
*Solanum indicum *L. (1043, Solanaceae)	Sabangang	Fruit	Dried fruits are boiled; decoction used to prepare pills (ca 10 g each) and is taken twice daily for curing high blood pressure (54%)
*Spilanthus paniculata *DC. (1059, Asteraceae)	Santustem	Flowers	Flowers (ca 200 g) are crushed and applied twice daily to relieve tooth ache and cure cavity formation (76%)
*Tabernaemontana divaricata *(L.) R. Br. (1146, Apocynaceae)	Santu-jri-iong	Latex	Latex is applied twice daily to prevent cavity formation (65%)
*Urena lobata *L. (1234, Malvaceae)	That-thu	Leaves	Decoction of the leaf is taken twice daily to reduce blood pressure; and also is taken before sleep to relieve rheumatic pain and body ache (69%)

The medicinal plant species used by the Jaintias were found to be distributed across 27 families and 37 genera. Different parts of medicinal plant species were used by them as medicine. For curing ailments, the use of aboveground plant parts was higher (76.59%) than the underground plant parts (23.41%). Of the aboveground plant parts, leaf was used in the majority of cases (23 species), followed by fruits (4). Different underground plant forms such as root, tuber, rhizome, bulb and pseudo-bulb have also been found to be in use as a source for curing ailments. The whole plant of 5 species [e.g. *Centella asiatica (L) Urban*, *Cuscuta reflexa *Roxb., *Oxalis corymbosa *L. and *Clerodendron serratum *(L) Moonb and *Scroporia dulcis *L.] were used as medicine. These 39 medicinal plant species were used in curing about 30 types of ailments, of which the highest numbers of plant species (20 species) were used for the treatment of gastrointestinal disorders such as indigestion and constipation. About 8 medicinal plant species were used in curing cough and cold, and 5 medicinal plant species were used for healing cuts and wounds (Table 1).

Different researchers from the country have reported altogether 2416 ethno medicinal uses of plants. Out of these, workers from the North East India itself have contributed to the knowledge of 1953 ethno medicinal uses of plants (Table 2). This whooping figure enriches the earlier report from this part of the country [3]. Different plants used by the Thottianaickans of Tamil Nadu, Miris of Assam, Nagas of Nagaland, tea tribes of Assam, Chakma community in Arunachal Pradesh, Meitei community in Manipur, Nishi tribes of Arunachal Pradesh, Mizo tribes of Mizoram, Monpas of Arunachal Pradesh, Mikirs of Assam, Shan tribes of Assam, *Khonds *of Andhra Pradesh, *Bhil *tribe in Madhya Pradesh, Apatani tribe of Arunachal Pradesh etc. [3,5,10-40] has some or the other relevance with the plants that are found to be in use by the Jaintia tribe residing in this remote part of India.

**Table 2:** Ethnobotanical uses of plants reported from different parts of India.

Tribes/Ethnic Groups/Indigenous people/Region	Number of plants reported	Authors
Apatani	158	Kala C P (2005)
Arunachal Pradesh	56	Tiwari K C et al (1996)
Arunachal Pradesh	464	Haridasan K et al (2002)
Assamese	35	Islam M (1996)
Bhil	62	Jadhav D (2006)
Cape Comorin	89	Jeeva S et al (2005)
Chakma	63	Sarmah R et al (2006)
Chellipale	51	Udayan P S et al (2005)
Dimasa	5	Dutta P K and Dutta B K (2000)
Dev Barma	8	Dutta P K and Dutta B K (2000)
Halam	3	Dutta P K and Dutta B K (2000)
Hmar	16	Dutta P K and Dutta B K (2000)
Jaintia	39	Sajem A L and Gosai K (present study)
Kaadar	41	Udayan P S et al (2005)
Karens	24	Sharief M U et al (2005)
Khasi, Jaintia	100	Kharkongor P and Joseph (1997)
Khonds	11	Rao V L N et al (2006)
Kuki	25	Dutta P K and Dutta B K (2000)
Malani	35	Sharma P K et al (2005)
Manipuri	4	Islam M (1996)
Meghalaya	56	Syiem D et al (1999)
Meghalaya	55	Kharduit J (1999)
Meitei	25	Dutta P K and Dutta B K (2000)
Meitei	20	Huidrom and Singh B K (1996)
Meitei	120	Khumbongmayum et al (2005)
Mikirs	24	Borthakur S K (1997)
Mishing	32	Hajra P K and Baishya (1997)
Mishing	44	Singh J et al (1996)
Mizo	17	Bhardwaj S and Gakhar S K (2005)
Mizoram	238	Lalramnghinglova J H (1996)
Monpas	15	Dam DP and Hajra P K (1997)
Munda, Santal		
Oraon, Polia	27	Mitra S and Mukherjee S K (2005)
Naga	14	Jamir N S (1999)
Naga	2	Islam M (1996)
Naga	26	Rao R R (1997)
Nishi, Apatani	154	Rawat M S and Choudhury S (1998)
Rongmei	20	Dutta P K and Dutta B K (2000)
Shan	8	Bora H R and Pandey A K (1996)
Tai Aiton, Tai Khamyang, Tai Turung and Sonowal Kachari	22	Pandey A K et al (1996)
Tea tribes	73	Das S et al (2000)
Thottianaickans	115	Ganesan S et al (2006)
Yobins	20	Yobin Y S H (1999)

The use of *Achyranthes aspera *L. against urinary disorders has been also reported amongst the Chakma community in Arunachal Pradesh [37] while the same species is used against eye burns in the Coastal region of Cape Comorin in India [38]**. **The root powder of *Asparagus racemosus *Willd also known as Shatavari has been found to be effective in chronic peptic ulcer [23] while the Jaintias use it for urinary disorders as well as stomach ache that could be due to high peptic juice secretion. *Cataranthus roseus *(L) G. Don, also known as an anti cancer drug yielding plant [39] too finds its usage in Arunachal Pradesh against diabetes. The use of *Centella asiatica *(L) Urban against stomach disorder is common to different tribes and communities of India [5,11,14,17,20,37] and [39] and is also used as a brain tonic [38]. Besides this, the inherent property of this plant to act against conjunctivitis and other eye injury has never been reported earlier. Similarly, the use of *Clerodendrum serratum *(L) Moonb against asthma has never been reported earlier; only its use against diverse form of skin diseases was found in the Coastal region of Cape Comorin in India [38]. *Ocimum sanctum *L. has a long Indian history of bearing an antitussive property but its analgesic use has never been reported earlier.

Thus it can be said now that the discovery of different plant species used by the Jaintias of North Cachar Hills district of Assam paves way the need to undertake a detailed ethnobotanical study of the whole hill districts of Assam involving as many tribes as possible. In spite of the rich wealth of bio-resources and potential, development is far from meeting the expectations of local people in Assam mainly in terms of existing health care facilities and herbal industries.

## Conclusion

The information generated from the present study regarding the medicinal plant use by the Jaintia tribes need a thorough phytochemical investigation including alkaloid extraction and isolation along with few clinical trials. This could help in creating mass awareness regarding the need for conservation of such plants and also in the promotion of ethno-medico-botany knowledge within the region besides contributing to the preservation and enrichment of the gene bank of such economically important species before they are lost forever.
